# Letter to Editor: Support to the Spanish language, from Oral Medicine

**DOI:** 10.4317/medoral.21742

**Published:** 2017-06-04

**Authors:** Eduardo Chimenos-Küstner

**Affiliations:** 1 Department of Odontostomatology, Oral Medicine. Hospitalet de Llobregat, Barcelona, Spain

## Abstract

In the form of a letter to the director, the author provides linguistic comments related to Spanish. He draws attention to some often misused words and suggests some expressions that may improve the use of the Spanish language in scientific texts.

** Key words:**Spelling, linguistics, scientific language.

## Dear Editor, 

I have read with great interest and satisfaction the LETTER TO THE DIRECTOR, published in the Spanish version of the Magazine, number 5, November 2016. It was signed by Professors José-Manuel Aguirre-Urizar and Adalberto Mosqueda-Taylor, whom I congratulate for their initiative. In this letter, the authors comment on the inappropriate use of some words, which are often translated from English into Spanish as desorden for “disorder”, verrucoso for “verrucous” or necrotizante for “necrotizing”. Against these terms, the authors propose (in my opinion, correctly) alternative versions: trastorno, verrugoso and necrosante, respectively (1).

As a contribution to the initiative of such illustrious professors, I would like to draw attention to some expressions, which should be taken into consideration when writing in Spanish. Although certain meanings used frequently are synonymous and correct [such as cardiaco/cardíaco (cardiac), periodo/período (period), cicatrizal/cicatricial (scarring)], the same is not true for certain words that are often used incorrectly (2-4). Some examples are given in  Table 1 in their correct and incorrect form.

**Table 1:**
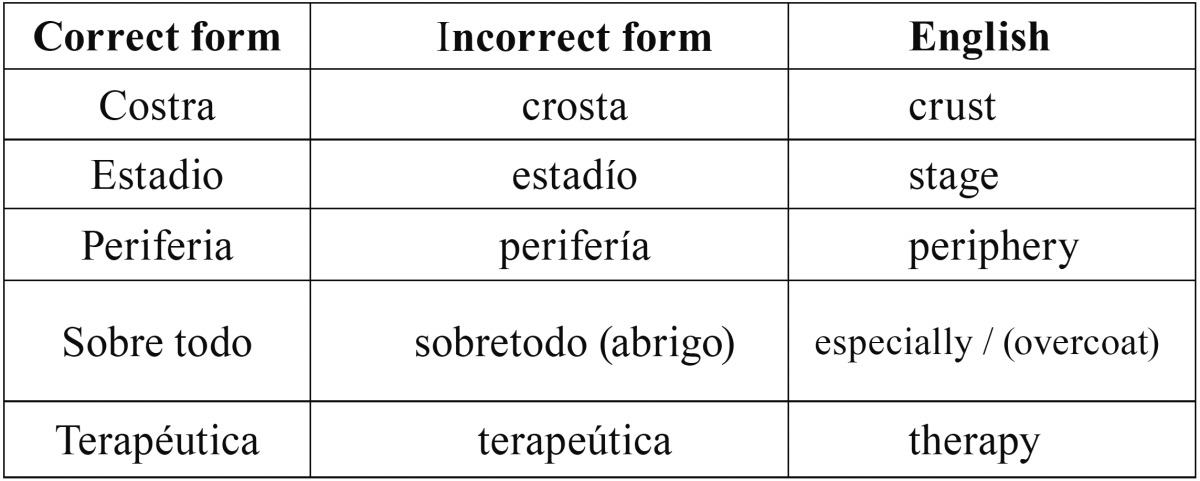
Frequent errors in writing in Spanish language (2-4).

On the other hand, there are correct terms, which are sometimes not used in the most appropriate way.  Table 2 contains some suggestions.

**Table 2:**
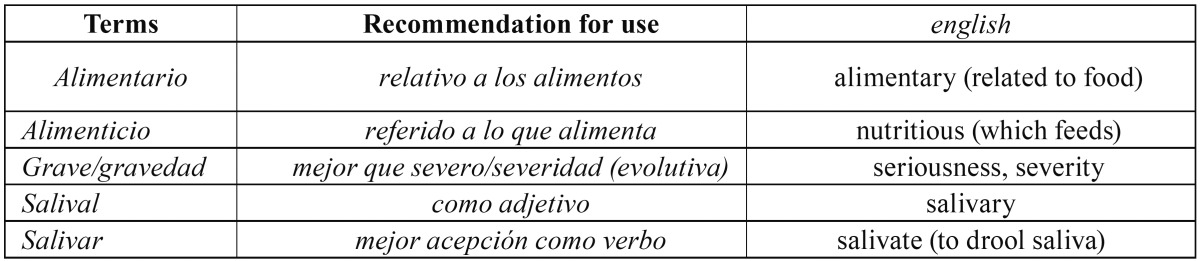
Alternative suggestions for some badly used terms.

Certain expressions, which are often used in scientific texts, could also be subject to discussion or debate, and may arouse interesting controversy. Among them.

- Condición or estado precanceroso (for precancerous condition)? 

- Leucoplasia/eritroplasia or leucoplaquia/eritroplaquia (for leukoplakia/erythroplakia)? 

- Patologías (Pathologies)? Would not be better: enfermedades, trastornos or alteraciones (diseases, disorders or alterations)? 

- Pieza dentaria (dental piece): is it not better diente (tooth)? 

- Recidivante or recurrente (recurrent)? 

Scientific language is a living being. It is a communication tool for those who use it. Therefore, as users we should contribute, suggest and debate, seeking to agree the most appropriate terminology in those areas were we are competent. Hopefully these comments encourage readers to contribute to a linguistic depuration of scientific language, enriching it with their contributions. Spanish is the vehicle language of a large number of people in the world and I think it is worth taking care of this language, as is done with others, also in the field of Science.

I would like to take this opportunity to express my gratitude and my deepest and most sincere congratulations for the magnificent and ascending editorial work achieved by the magazine under your direction. It has become a great reference in the field of health, in its more than 20 years of history.

Thank you very much.
